# Readmission after TIPS: an up-to-date landscape

**DOI:** 10.1093/gastro/goz063

**Published:** 2019-11-28

**Authors:** Octavi Basssegoda, Andres Cardenas

**Affiliations:** 1 GI/Liver Unit, Institut de Malalties Digestives i Metaboliques, Hospital Clínic, University of Barcelona, Barcelona, Spain; 2 Institut D’investigacions Biomèdiques August Pi I Sunyer (IDIBAPS), Centro de Investigación Biomédica en Red de Enfermedades Hepáticas y Digestivas (CIBEReHD), Barcelona, Spain

Portal hypertension in patients with cirrhosis is common and can lead to severe complications that are associated with decreased survival. Among these complications, portal hypertensive bleeding and refractory ascites in many cases can be managed with the placement of a transjugular intrahepatic portosystemic shunt (TIPS). Current indications of TIPS placement include variceal hemorrhage refractory or recurrent to standard therapy with vasoactive drugs and endoscopic variceal ligation [1]. Also, in carefully selected patients (i.e. Child–Pugh class C cirrhosis with score 10–13 and Child Turcotte Pugh (CTP) class B with active bleeding on endoscopy), early TIPS (placed within 72 hours of admission) after vasoactive drugs and endoscopic band ligation improves outcomes. This intervention reduces the risk of rebleeding among this group of high-risk patients and is associated with increased survival [2]. TIPS is also a treatment of choice in patients bleeding from cardiofundal varices (GOV2 and IGV1) and ectopic varices [1]. Refractory ascites is also an indication for TIPS placement. However, its efficacy is controversial in this setting. It is clear that TIPS is associated with a better control of ascites than large-volume paracentesis. That said, TIPS is followed by a greater incidence of hepatic encephalopathy. Controversial results regarding the survival benefits of TIPS have emerged and are still a matter of intense debate [3]. In summary, a careful selection of candidates for TIPS placement is necessary if refractory ascites is the indication for TIPS. Specifically, TIPS can be detrimental in older patients with cardiopulmonary disease as well as in patients in CTP class C and higher Model for End Stage Liver Disease (MELD) scores. It has to be noted that most of the randomized trials evaluating survival of TIPS have been performed using bare stents [4].

Complications of TIPS placement are well known and can be divided in intraprocedural complications (acute hemorrhage, non-target TIPS insertion, or puncture), early post-procedural (acute hepatic encephalopathy, acute hepatic failure secondary to liver ischemia, biliary complications, TIPS migration, and occlusion) and delayed complications (recurrent portal hypertension and infection) [5]. A major and well-known complication of TIPS placement is the development of hepatic encephalopathy. In the era of uncovered stents, this complication occurred in up to 55% of patients [6]. In 2004, the new polytetrafluoroethylene (PTFE)-covered stents were approved by the Food and Drug Administration. It was thought that the increased patency of these new stents and the lower risk of shunt dysfunction would be associated with a greater incidence of hepatic encephalopathy. However, a similar incidence of this complication was found with the new stents [7]. Factors associated with the development of hepatic encephalopathy after TIPS placement are older age, advanced liver failure, and a history of previous hepatic encephalopathy [8]. There is promising data indicating that rifaximin can prevent hepatic encephalopathy after TIPS [9].

In this issue, Dr Vozzo and colleagues performed a unicentric retrospective analysis between 2004 and 2017 pointing at identifying the 30-day readmission rate after primary TIPS placement and to assess its potential predictive factors. All TIPS were covered stents [10]. The study included all patients in whom TIPS was placed for any indication and analysed the 30-day all-cause readmission rate in this cohort of patients. They analysed a large retrospective cohort of 566 patients and found an overall 30-day readmission rate of 36%. Half of the readmissions were secondary to hepatic encephalopathy. Other less common causes of readmission were infection (15%), bleeding (11%), and fluid overload (7%). The only factor independently associated with readmission was the MELD score. Also, an important point is that the 90-day survival was significantly decreased among patients who were readmitted 30 days after TIPS placement.

The study refers to an important and not well-studied question. Rowley *et al.* [11] studied the risk of hepatic encephalopathy hospital-readmission rate after TIPS placement but no data exist on other readmission causes. Rush *et al.* [12] studied the 90-day readmission rate after TIPS placement only in patients who had TIPS placed because of variceal bleeding. In this case, TIPS placed for any indication were analysed. As has been shown in this study, the two main indications were refractory ascites and variceal bleeding. The authors did not find significant differences in readmission rates between patients with previous history of hepatic encephalopathy and those without, although a trend for significance was present (odds ratio 1.26, 95% confidence interval: 0.95–1.95, *P* = 0.092). An interesting question raised by this article is whether patients should receive prophylactic lactulose treatment after TIPS placement. The authors analyse this fact, comparing patients without history of hepatic encephalopathy who received prophylactic lactulose vs patients who did not. The prophylactic treatment was administered depending on the discharging provider’s discretion and not according to a unified protocol. Only half of patients in both groups received lactulose treatment at discharge and only one-third of patients were followed up by a hepatologist within 2 weeks. As this is a non-controlled study, it is limited by the possibility of bias interpretation of these results. However, they did not find any significant difference in readmission rates among both groups. Controlled studies are needed to further clarify this fact. A randomized–controlled trial exists on this matter, but no data exist in the era of covered stents [9].

The study has several strengths. First, a large number of TIPS are analysed and no exclusion according to indication for TIPS was done. All indications were included, but most were due to variceal bleeding and refractory ascites. The exclusion of non-covered stents, which are no longer used, provides some external validity. Second, a more advanced hepatic dysfunction is associated with worse outcomes after TIPS placement, as we have already mentioned. In this study, a large proportion of advanced patients were included. Specifically, 62 patients with a MELD score of 20–30 were included. Among those, 58% were readmitted at 30 days as compared to a 33% readmission rate of patients with a MELD of <20. Clinicians should keep in mind those numbers when indicating TIPS for patients with advanced liver disease.

In conclusion, this large retrospective unicentric study gives us an up-to-date landscape of readmissions after TIPS placement including all TIPS indications and many advanced patients. More studies are needed to help manage and prevent those complications in order to avoid readmissions.

## Conflicts of interest

None declared.
